# Bionanocomposite Films Containing Halloysite Nanotubes and Natural Antioxidants with Enhanced Performance and Durability as Promising Materials for Cultural Heritage Protection

**DOI:** 10.3390/polym12091973

**Published:** 2020-08-31

**Authors:** Giulia Infurna, Giuseppe Cavallaro, Giuseppe Lazzara, Stefana Milioto, Nadka Tzankova Dintcheva

**Affiliations:** 1 Department of Engineering, University of Palermo, Viale delle Scienze, Ed. 6, 90128 Palermo, Italy; giuliainfurna92@gmail.com; 2 National Interuniversity Consortium of Materials Science and Technology (INSTM), Research Unit of Palermo, Via G. Giusti, 9, 50121 Florence, Italy; giuseppe.cavallaro@unipa.it (G.C.); giuseppe.lazzara@unipa.it (G.L.); stefana.milioto@unipa.it (S.M.); 3 Department of Physics & Chemistry, University of Palermo, Viale delle Scienze, Ed. 17, 90128 Palermo, Italy

**Keywords:** antioxidants, biopolymer blends, chitosan, halloysite nanotubes, pectin

## Abstract

In the last decade, the interest toward the formulation of polymer films for cultural heritage protection continuously grew, and these films must be imperatively transparent, removable, and should not react/interact with surface of the artworks. In this research, bionanocomposite films, based on chitosan (Ch) and pectin (P) and containing naturally occurring fillers and antioxidants, were formulated by solvent casting methods and were accurately characterized. The natural halloysite nanotubes (HNT) have a two-fold role, specifically, physical compatibilizer and antioxidant carrier. Therefore, the theoretical solubility between Ch and P was estimated considering Hoy’s method for solubility of polymers, while the optimum ratio between biopolymer constituents was assessed by ζ-potential measurements. The transparency, wettability, and mechanical behavior of Ch:P films, also in presence of HNT without and with antioxidants, were investigated. The beneficial effects of natural antioxidants, such as vanillic acid (VA) and quercetin (Q), on Ch:P/HNT durability were found.

## 1. Introduction

In the last decades, the interest in maintenance and re-construction of the world’s cultural heritage through introduction of protective polymer films/coatings has constantly grown and as expected, these films/coatings must be imperatively transparent, removable, chemically compatible, and not able to interact/react with protected surface. Therefore, advanced materials based on acrylate and vinyl polymers are usually considered to formulate efficient protective organic films/coatings for cultural heritage conservation and protection. Specifically, there are (i) Paraloid B72^®^, based on ethyl metacrylate/ethyl acrylate copolymer; (ii) Mowilith DM5^®^ and Mowilith DMC2^®^, based on vinyl acetate/n-butyl acrylate copolymer and vinyl acetate/dibutyle maleate copolymer, respectively; and (iii) Axilat series^®^, based on styrene/acrylic copolymers. Main advantages in considering protection films/coatings based on acrylates, methacrylates, and vinyl groups are their good transparency, barrier properties towards oxygen, pollutants, moisture, UV-light, and outstanding mechanical resistance, but unfortunately, due to the presence of numerous intrinsic carbonyl groups and instaurations, these polymer materials are subjected to untimely yellowing, showing limited durability [1,2,3,4]. 

Currently, a well-defined trend is the moving from synthetic polymers to naturally occurring counterparts, also considering their eco-friendly and non-toxic nature. It is worth noting that the introduction of bio-based materials in different application sectors, such as food packaging, daily disposables, pharmaceutical sector, and biomedical device, is currently a consolidate practice also at large scale. Additionally, bio-based materials are considered also good candidates for unconventional applications in automotive, transports, building, and construction sectors [5,6,7,8], and nowadays, for cultural heritage protection [9,10,11]. Moreover, according to current literature, the minimization of the ecological and environmental impact of materials is the driving effort for the development of new innovative materials for various end-use applications. As known, both the synthesis of biopolymers using natural monomers and the manufacturing of biopolymer-based materials containing only natural additives have gained great attention in the last two decades, especially considering the production of end-use applications ranging from automobile to the building industries. It is worth noting that the replacement of fossil-based polymers by bio-based counterparts is possible if the properties and performance (for example, mechanical resistance, barrier properties, thermo- and photo- oxidation stability, and long-time durability of the biomaterials) are competitive to that of synthetic materials. To this end, the research and introduction of naturally occurring additives that can enhance the biopolymers properties and performance is absolutely required [12,13,14,15].

Therefore, biopolymers, such as polyesters, polysaccharides, protein, lipids, starch, alginate, and cellulose, are excellent candidates for replacing numerous polyolefins. According to current literature, the bio-polysaccharides are non-toxic, biocompatible, renewable, and have also reasonable film-forming ability [6,7]. Additionally, bio-polysaccharides, such as chitosan (Ch) and pectin (P), are abundant in nature, relatively cheap materials, and excellent candidates for food packaging, considering their ability to form edible food-compatible coatings and guarantee excellent food safety [16].

Chitosan (Ch) is a chitin derivative, which is the most abundant polysaccharide on earth, after cellulose, and, for instance, the chitin is present in the cell wall of fungi or in the shell of arthropods such as crustaceans (i.e., crabs, shrimps, or lobsters). Chitosan is a cationic polysaccharide, obtained through the controlled deacetylation of chitin in the presence of NaOH, and it is mainly a linear polymer of β-(1-4)-linked d-glucosamine (deacetylated unit) and N-acetyl-d-glucosamine (acetylated unit). Chitosan, if added as additive to biopolymers, can effectively improve the mechanical property and barrier property of biopolymer film, and additionally, it possesses the capacity to adjust the release rate of active film ingredients, enlarging its action time and effect [17,18,19,20].

Pectin (P), formed with D-galacturonic acid linking by α-1, 4 glycosidic bonds, is a typical linear heteropolysaccharide [21,22]. Pectin is widely considered as bio-coating film for fruits and food due to its biodegradability, biocompatibility, safety, and edibility. Pectin is an anionic polysaccharide, because of the presence of numerous negatively charged carboxylic groups in its chemical structure, and for this reason, it shows the tendency to interact with metal cations and active ingredients. An exciting study by Fraeye et al. [23] documents that the pectin may form a three-dimensional network structure by adding bivalent cations, such as calcium ions, and the calcium ions may be packed in the interstices of the twisted pectin chains, which may improve the film’s properties.

Therefore, biopolymers, such as Ch and P, can be considered good candidates to formulate biopolymer blends for the development of edible coatings and films, also at an industrial scale, because of their biodegradability, non-toxicity, and film-forming ability [24,25,26]. An interesting study investigated the best processing conditions for Ch:P blends, produced through a casting method [20]. Another interesting study documented the possibility to form edible solvent-casting Ch:P films using citric acid as cross-linking agent [25]. Both studies concluded that Ch:P blend films are good candidates for the formulation of edible packaging materials, optimizing the final properties also through the use of a suitable naturally occurring cross-linking agent.

The introduction of natural additives, such as reinforcement agents, cross-linking agents, and antioxidants, to single biopolymers or biopolymer blends favors the formulation of sustainable, eco-friendly, and non-toxic materials, having a beneficial effect on human health and the environment [27]. Among natural fillers, halloysite represents an emerging nanomaterial for the fabrication of bionanocomposites useful in numerous applications, such as food packaging [28,29,30], tissue engineering [31,32,33,34], delivery systems [35,36,37,38], photo-protection [39,40], and restoration [41,42]. As examples, the filling of pectin with halloysite nanotubes containing rosemary essential oil generated nanocomposite films with antimicrobial and antioxidative functionalities [28], while the combination of chitosan and halloysite loaded with diclofenac was exploited to fabricate tablets with controlled release properties [37]. Halloysite possesses a hollow tubular morphology with a length ranging from 100 to 2000 nm, whereas the intervals for the external and internal diameters are 20–150 and 5–70 nm, respectively. As demonstrated by electron microscopies [43] and scattering techniques [44], the size polydispersity of the clay nanotubes is influenced by their specific geological source. Due to its large specific surface (28–80 m^2^·g^−1^), halloysite was extensively employed as a natural support for catalysis applications [45,46,47], as well as an adsorbent nanomaterial for wastewater remediation [48,49,50,51]. It should be noted that halloysite is a biocompatible nanoparticle with a low toxicity, which was proved by both in vitro and in vivo investigations [52,53,54].

However, the immobilization through chemical linkage or physical absorption of antioxidant molecules onto the surface of nanoparticles, i.e., using the particles as carrier for antioxidant molecules, is a new approach for “smart” efficient stabilization of polymer and biopolymer-based nanocomposites, obtaining materials with enhanced durability [55,56,57,58]. Applying this approach, the antioxidant functionalities act at the interface between the inorganic and organic phase, which is the critical zone for the begging of the degradation processes of polymer- and biopolymer-based nanocomposites.

In this research, Ch:P films, containing halloysite nanotubes (HNT) and antioxidant (AO), were successfully formulated by the solvent-casting technique. The HNT have a two-fold role in Ch:P blends, specifically, they act as physical compatibilizer for the polymer blends and as carrier for antioxidant molecules (AO), such as vanillic acid (VA) and quercetin (Q). To establish the theoretical solubility between Ch and P, Hoy’s method was invoked, while their optimum ratio was estimated through ζ-potential measurements. The effect of HNT and AO presence on film transparency and optical properties was evaluated through spectroscopic analysis. Finally, the effect of HNT and AO on mechanical behavior, wettability, and photo-oxidative resistance of Ch:P films was studied and evaluated considering these bionanocomposite films as promising materials for cultural heritage protection.

## 2. Materials and Methods 

### 2.1. Materials

The materials used in this research are

- Chitosan (Ch), low viscosity, deacetylation degree = 75–85% and average molecular weight = 120 kg·mol^−1^;
- Pectin (P) from Citrus, Poly-D-galacturonic acid methyl ester with degree of methyl esterification 24%, Mw = 30–100 kg·mol^−1^;
- Halloysite nanoclay (HNT), [Al_2_Si_2_O_5_(OH)_4_] × 2H_2_O, formula weight = 294.19 g·mol^−1^; pore volume = 1.26–1.34 mL/gm; pH = 4.5–7.0; diameter = 30–70 nm; length = 1–3 microns;
- 4-Hydroxy-3-methoxybenzoic acid, named vanillic Acid (VA), Molecular Weight = 168.15 g·mol^−1^;
- 2-(3,4-Dihydroxyphenyl)-3,5,7-trihydroxy-4H-1-benzopyran-4-one hydrate, named quercetin (Q), molecular weight = 302.24 g·mol^−1^ (anhydrous basis).

All used materials (i.e., Ch, P, HNT, VA, and Q) were purchased by Sigma-Aldrich (Milan, Italy).

### 2.2. Loading of Halloysite Nanotubes with Antioxidants

Solutions of VA (2% wt./wt.) and Q (0.2% wt./wt.) in ethanol were prepared and equilibrated overnight. Halloysite nanotubes, as a dry powder, were added to the ethanol solution of antioxidant (VA/HNT and Q/HNT mass ratios 1:5). The vacuum pumping strategy to enhance the loading efficacy into the halloysite nanotubes is described elsewhere [59]. The efficiency of loading was verified by means of thermogravimetric analyses (TGA; see Figure S1 in Supplementary Materials). The experiment was carried out by using a Q5000 IR apparatus (TA Instruments) under nitrogen flow (25 cm^3^/min) at a heating rate of 20 °C/min to 800 °C. The amount of antioxidant entrapped into the HNT was estimated from the residual mass at 750 °C taking into account for the residual mass of each component and assuming the rule of mixtures. The thermogravimetric curves are provided in the supporting files and the obtained loading efficacy expressed as antioxidant mass percent into the composite with HNT are 0.537% *w*/*w* and 2.59% *w*/*w* for HNT/VA and HNT/Q, respectively.

### 2.3. Preparation of Bio-Nanocomposite Films

Aqueous solutions of each biopolymer at 2% wt. were prepared following the procedure detailed elsewhere and equilibrated overnight [37,60]. Then, an appropriate amount of halloysite nanotubes (HNTs) was added to the polymer solution or to a mixture of the two polymer solutions and kept under stirring overnight. The well-dispersed aqueous mixture was poured into glass Petri dishes (15 g) under vacuum at room temperature to evaporate water until the weight was constant and to obtain biofilms with a thickness ranging from 80 to 130 μm.

### 2.4. Characterizations

#### 2.4.1. ζ-Potential and Isoelectric Point

The ζ-potential was obtained from electrophoretic mobility measured by a Zetasizer Nano-ZS (Malvern Instruments, Malvern, UK) at 25.0 ± 0.1 °C by using a disposable folded capillary cell.

#### 2.4.2. FT-IR Analysis

A Fourier Transform Infrared Spectrometer (Spectrum One, Perkin Elmer, Shelton, CT, USA). Shelton, CT, USA). was used to record IR spectra using 16 scans at a resolution of 1 cm^−1^. Measurements were obtained from the average of triplicate samples with a calculated maximum experimental error (relative standard deviation) of around 5%.

The progress of degradation for Ch:P and Ch:P-based systems was followed by running FTIR analysis with time and monitoring the variations in the range of 1700–1480 cm^−1^ in time, using the Spectrum One software. The deconvolution of FTIR peaks was performed using the scientific software OriginPro 2015 (Northampton, MA, USA).

#### 2.4.3. UV–visible Analysis

A UV–visible spectrometer (Specord^®^250 Plus, Analytikjena, Torre Boldone, BG, Italy) was used to record UV–Vis spectra performing 8 scans between 200 and 1100 nm at a resolution of 1 nm.

#### 2.4.4. Contact Angle Measurements

The water contact angle was measured by means of an OCA 20 (Data Physics Instruments, Filderstadt, Germany) apparatus equipped with a CCD camera and a high-performance digitizing adapter. The SCA 20 software (Data Physics Instruments) was used for data acquisition. The films were fixed on top of a plane solid support and kept flat during water deposition and acquisition. The sessile drop method was used with a droplet volume of 6 μL. Temperature was controlled for the support and the injecting syringe at 25.0 ± 0.1 °C. A total of 5 droplets were examined for each film sample. 

#### 2.4.5. Mechanical Characterization

Tensile tests were carried out using a Universal Testing Machine (Instron model 3365, Bucks, UK), following the ASTM D882 method, on rectangular samples cut by films prepared by solvent-casting. The tests were performed, using a tensile speed at 1 mm/min for 1 min in order to evaluate the Young’s Modulus, and then the velocity was increased to 10 mm/min until sample breakage.

#### 2.4.6. Optical Observations

The optical micrographs were taken with an Optika polarizing microscope at room temperature.

### 2.5. Photo-Oxidation Exposure

Photo-oxidation of Ch:P and Ch:P-based films (about 80 μm thick) was carried out using a Q-UV-Solar Eye weatherometer (from Q-LAB, Westlake, OH, USA) equipped with UVB lamps (340 nm). The weathering conditions were a continuous light exposure at T = 55 °C.

### 2.6. Solubility Theoretical Calculation

The theoretical solubility parameters for both biopolymers, i.e., Ch and P, were calculated following Hoy’s method [61] as follows:
Expressions for δ and δ-components$\delta_{tot}=\left(F_{i}+B/\bar{n}\right)/V$, **B** = 277$\delta_{p}=\delta_{tot}{\left(\frac{1}{\alpha^{\left(P\right)}}\frac{F_{p}}{F_{t}+B/\bar{n}}\right)}^{1/2}$, $\delta_{h}=\delta_{tot}{\left[\left(\alpha^{\left(P\right)}-1\right)/\alpha^{\left(P\right)}\right]}^{1/2}$, $\delta_{d}={\left(\delta_{tot}^{2}-\delta_{p}^{2}-\delta_{h}^{2}\right)}^{1/2}$,Additive molar functionsF*_t_* = ΣN*_i_*F*_t,i_*, F*_p_* = ΣN*_i_*F*_p,i_*, V = ΣN*_i_*V*_i_*, Δ_T_
*^(P)^* = ΣN*_i_* Δ_T T,i_
*^(P)^*,Auxiliary equationsα*(P)* = 777 Δ_T_*^(P)^*/V, *n* = 0.5/Δ_T_*^(P)^*,
where:

F*_t_* is the molar attraction function and F*_p_* its polar contribution;
V is the molar volume of the solvent molecule or the structural unit of the polymer;
ΔT is the Lyndersen correction for non-ideal behavior, α is the molecular aggregation number;
*n* is the number of repeating units per effective chain segment of the polymer;
*δ_tot_* is the solubility parameter;
*δ_p_* is the contribution of the polar forces;
*δ_h_* is the contribution of the hydrogen bonding;
*δ_d_* is the contribution of the dispersion forces.

## 3. Results and Discussion

### 3.1. Theoretical Solubility and z-Potential Estimation

To evaluate the solubility of P in Ch, Hoy’s solubility theory was invoked. Particularly, the theoretical solubility parameters for both biopolymers, i.e., Ch and P, were calculated following Hoy’s method [61], using the formulas reported in Section 2.6. Considering all functional groups presented in both Ch and P monomers, the calculated values of theoretical solubility parameters are shown in Table 1.

Generally, based on Hoy’s method, for a good solubility between two different organic components, having different chemical natures and containing different organic groups, the difference between their solubility parameters must be ≤5 (J/cm^3^)^1/2^. It is worth noting that based on the calculations, the difference between the solubility of Ch and P parameter is |∆| = 1.28 (J/cm^3^)^1/2^, and this result highlights a very good miscibility between these two biopolymers.

**Figure 1:**
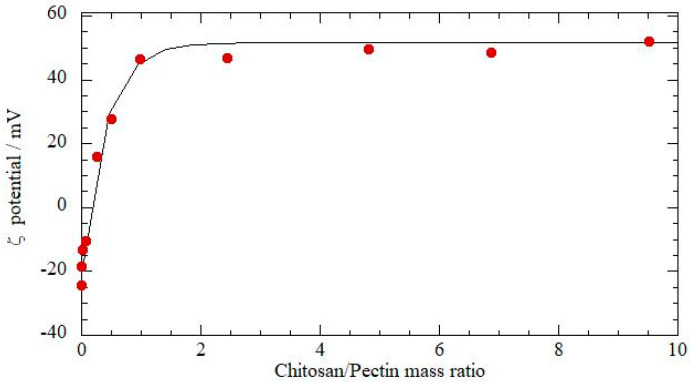
The ζ-potential for Ch:P blends at a different ratio.

However, considering the very good theoretical solubility of P in Ch and to formulate biopolymer-based films suitable as protective coverages, the ζ-potential measurement was carried out to estimate the constituent optimal ratio, i.e., Ch:P ratio. In particular, we thought it would be interesting to exploit the possibility to prepare a complex blend with the two biopolymers. On the one hand, the charge neutralization should ensure a film with less leaching issues upon water exposure, but as soon as the ζ-potential values are below a given threshold, a low colloidal stability and phase separation occurs. With this in mind, the ζ-potential was determined from electrophoretic mobility measurements on Ch:P solutions with a variable Ch:P mass ratio, see Figure 1. As expected, the ζ-potential values are negative in excess of pectin, while they turn to positive values when chitosan is added. The values extrapolated for pure polymers agree with the literature reports on aqueous pectin or chitosan solution [62]. The null charge that corresponds to the matching between positive chitosan and negative pectin is obtained at a Ch:P mass ratio equal to 0.13. It should be noted that at this peculiar mass ratio, the casted films were not homogeneous, presenting phase separation and precipitation in the casting mixture. From data in Figure 1, it can be estimated that a Ch:P mass ratio equal to 1 is the most suitable composition to have a good colloidal stability of the polymers in water. As a matter of fact, homogeneous film blends have been observed under these circumstances.

### 3.2. Spectroscopy and Contact Angle Analysis 

To formulate biopolymer-based films that can be considered for cultural heritage protection, the Ch:P was added with halloysite nanotubes (HNT) and natural antioxidants, such as vanillic acid (VA) and quercetin (Q). In this case, HNT has a dual role in the Ch:P blend, specifically, physical compatibilizer and antioxidant carrier; both VA and Q were immobilized onto HNT, as accurately explained in the experimental section. Therefore, according to current literature, the physical immobilization of antioxidant molecules onto inorganic nanoparticles is an innovative approach in stabilizing polymer-based nanocomposites [54]. The action of antioxidant molecules at the interface between inorganic nanoparticles and polymer macromolecules has a beneficial effect on the performance and long-term durability of nanocomposites, considering that the interface area is a critical zone for the beginning of nanocomposites degradation.

**Figure 2:**
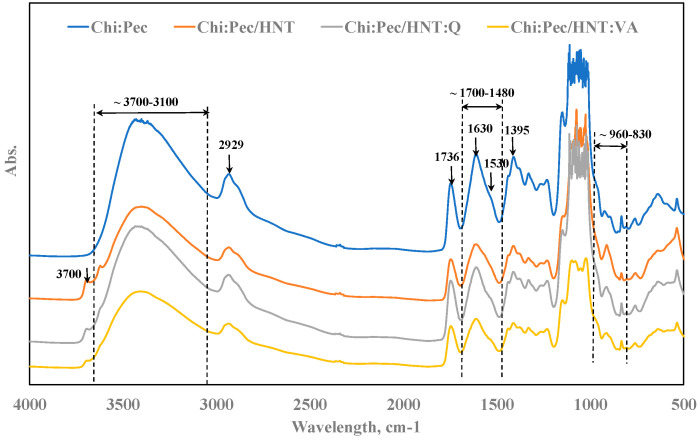
FTIR spectra of Ch:P and Ch:P containing natural halloysite nanotubes (HNT) without and with natural antioxidants.

To investigate the effect of added HNT to the Ch:P blend, accurate FTIR and UV–visible spectroscopy analyses were performed. In Figure 2, FTIR spectra of neat Ch:P and Ch:P containing HNT, HNT/VA, and HNT/Q are plotted, and as noticeable, all spectra are similar. However, according to literature, in Table 2, main characteristic absorption bands in the spectra of Ch and P are assigned [17,21,24,25].

A large absorption band in the range of 3700–3100 cm^−1^ is observed in the Ch:P spectrum, see top (blue) spectrum in Figure 2, and this can be attributed to the presence of numerous free and coordinated hydroxyl groups in polysaccharide structures. Additionally, this absorption band in the spectrum of Ch:P/HNT, and even more of Ch:P/HNT:Q and Ch:P/HNT:VA, appears much lower than that of Ch:P, probably because there are lower amounts of coordinated hydroxyl groups. It is worth noting that a small shoulder, approximately at 3700 cm^−1^, in the spectra of Ch:P/HNT, Ch:P/HNT:Q, and Ch:P/HNT:VA appears, and this shoulder can be attributed to the presence of coordinated hydroxyl groups in the alumino-silicate structures [42]. 

A well-visible absorption peak at around 1736 cm^−1^ is noticeable in Ch:P spectra, and there is a complex peak due to stretching of ester groups in Ch (at 1736 cm^−1^) and esterified carboxylic groups in P (1739 cm^−1^). The absorption peaks due to the presence of carboxylic anions (at 1630 cm^−1^) appear significantly shifted and influenced by the presence of HNT. Similar considerations can be made also for absorption bands at 1530 cm^−1^, related to the asymmetric vibration of N–H (N-acetylated residues of the amide II band), at 1395 and 1353 cm^−1^, assigned to N–H in plan deformation and C=N stretching (amide III band), respectively. 

Therefore, due to the presence of HNT, and even more the presence of HNT:Q and HNT:VA, the hydroxyl absorption band appears much lower and both peaks at 1630 cm^−1^ and 1530 cm^−1^ shift towards a lower wavelength. These effects can be understood considering that the presence of alumino-silicates nanoparticles modifies the biopolymers constituents’ interactions, i.e., it influences the interactions between carboxylic anions and N–H groups, and according to literature, these nanoparticles can act as physical compatibilizers for biopolymer blends [27].

**Figure 3:**
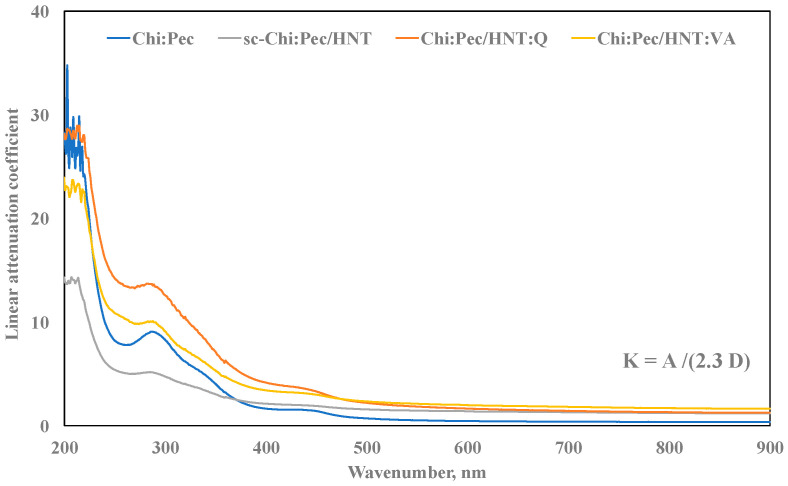
Linear attenuation coefficient (K) of Ch:P and Ch:P containing HNT without and with natural antioxidants.

Different small absorption bands in all spectra in the range of 960–830 cm^−1^ are observed, see Figure 3, and they can be assigned to the presence of specific intrinsic groups in Ch and P, listed in Table 2 as well. Therefore, according to literature [42], in FTIR spectra of alumino-silicates appear peaks at around 910 cm^−1^ and a small shoulder at around 3700 cm^−1^ (discussed above), due to metal-oxide structures and coordinated hydroxyl groups, respectively. Really, the absorption band at 910 cm^−1^ is partially overlapped by the presence of different small peaks in this range (950–890 cm^−1^) due to intrinsic structures of both polysaccharides (see assignment of IR absorption bands in Table 2). 

To sum up, all spectra, shown in Figure 2, are very similar to each other because of different reasons, specifically: (i) numerous absorption bands are overlapped due to the similarity of groups presented in the structure of both Ch and P; (ii) HNT act as physical compatibilizer and their presence modifies the interaction between Ch and P polar groups; (iii) the assignment of VA and Q characteristic peaks in these complex spectra is a hard matter because of overlapping by Ch and P peaks and their low concentrations. 

To evaluate the suitability for cultural heritage protection of Ch:P blends, containing HNT without and with antioxidant molecules, UV–visible analysis was performed, and in Figure 3, the trends of a linear attenuation coefficient as a function of the wavenumber are plotted. The values of linear attenuation coefficients (K) were calculated considering the measured absorption values (A) and sample thickness (D), using the formula reported in the figure.

Considering the UV range (200–380 nm), the values of linear attenuation coefficients of Ch:P/HNT are lower than the values of unfilled Ch:P, and interestingly, the values are significantly higher due to the presence of VA and Q molecules, suggesting a beneficial effect due to the presence of the antioxidant molecules.

Concerning the visible range (380–800 nm), as known, there is a very important range for film transparency, which is determinant for the applications of films as cultural heritage protective coverages. The values of linear attenuation coefficients in the visible range of Ch:P/HNT, also with both VA and Q, are slightly higher than the obtained values for the Ch:P blend, and really, these variations are negligible, and fortunately, the films are almost transparent upon HNT adding and suitable for cultural heritage protection.

Therefore, to evaluate the influence of HNT presence on film wettability, contact angle measurements were performed, and the obtained results are shown in Table 3 for the water droplet just after deposition. The good droplet symmetry is proved by the coincidence of left and right contact angle values. The contact angle for the blend is consistent with the contact angle values reported for pectin and chitosan pristine polymers, which present a similar value [63,64]. It is worth noting that the presence of HNT generates a slight decrease of the water contact angle values. The change in contact angle values is sensitive to the surface roughness and the chemical hydrophilicity of the film. In this case, the presence of the hydrophilic clay at the interface is consistent with the observed changes. It should be noted that the presence of Q and VA in the halloysite nanotubes does not further alter the film wettability.

### 3.3. Mechanical Behavior and Optical Observations

**Figure 4:**
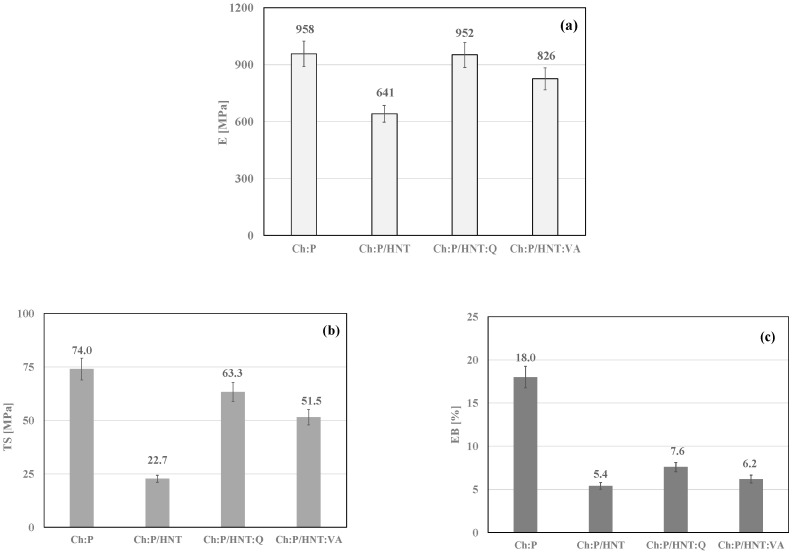
Main mechanical properties: (**a**) elastic modulus, E, (**b**) tensile strength, TS, and (**c**) elongation at break, EB, of Ch:P and Ch:P/HNT without and with natural antioxidants.

To evaluate the mechanical behavior of Ch:P blends, the films were subjected to a tensile test and the obtained results of the elastic modulus (E), tensile strength (TS), and elongation at break (EB) are plotted in Figure 4a–c. It is worth noting that the HNT adding to Ch:P leads to a decrease of the E value, probably because the HNT presence increases the heterogeneity significantly at a high concentration, i.e., 20% wt., see Figure 4a. Interestingly, due to the adding of HNT:VA, and even more HNT:Q, the system rigidity is significantly higher than the Ch:P/HNT, highlighting a beneficial effect of the VA and Q presence. Similarly, both TS and EB values decrease by HNT adding, see Figure 4b,c, respectively, while due to the presence of HNT:VA and HNT:Q, the properties at break increase in comparison to that of HNT. The latter suggests that the samples become more ductile due to the VA and Q presence, and this could be understood considering that the low molecular weight molecules, such as naturally occurring antioxidants VA and Q, exert a well-pronounced plasticizing effect, which is in accordance with the literature [27,65,66].

The film morphology was observed under an optical microscope to evidence the surface characteristic. The structure observation of biopolymers through high energy analysis, such as SEM and TEM, is a hard matter because, as known, these materials are susceptibility to fast degradation under a high energy beam [27]. Therefore, the Ch:P blend at the neutralization mass ratio (Ch:P = 0.13) clearly evidenced the presence of some fiber-like structures, while casting the more stable dispersion at Ch:P = 1 generates a very homogeneous film, see Supplementary Figure S2a,b. The nanocomposite film with halloysite nanotubes was still homogeneous, see Supplementary Figure S2c. It should be noted that the halloysite amount of 20% wt. is at the limit of the percolation [60] concentration. Therefore, the nanotubes amount was chosen at the maximum value that does not generate a significant halloysite clustering.

### 3.4. Photo-Oxidation Resistance

To evaluate long-term durability of Ch:P blends, thin films were subjected to accelerated UVB exposure. The photo-oxidative degradation in time of Ch:P, Ch:P/HNT, Ch:P/HNT:VA, and Ch:P/HNT:Q films was monitored through FTIR analysis at regular intervals of about 8 h. However, according to literature, the Ch degradation mechanism occurs mainly by depolymerization, followed by deacetylation, oxidation, and interchain cross-linking [67], while P depolymerization proceeds mainly by backbone hydrolysis, β-elimination, and decarboxylation [68].

As discussed above and according to literature, the Ch:P spectrum presents numerous multiple peaks, some of them overlapped, and considering the complexity of degradation phenomena for both Ch and P, the investigation of photo-oxidation resistance of Ch:P films through FTIR analysis is a hard matter; obtained spectra of all investigated bionanocomposite films are reported as supplementary materials, see Figures S3–S6. Therefore, it is chosen to follow the photo-oxidative degradation of Ch:P monitoring the changes in the spectra in a range of 1700–1480 cm^−1^ in time, which contains two different peaks; first peak at around 1630 cm^−1^ due to the presence of C=O of ion COO stretching of secondary amide group (amide I) for Ch (~1626 cm^−1^) and –O^(−)^–C=O for P (~1634 cm^−1^), and expectedly, due to C=O stretching for both Ch and P, a single peak and a second peak appear at around 1530 cm^−1^ attributed to N–H bending.

**Figure 5:**
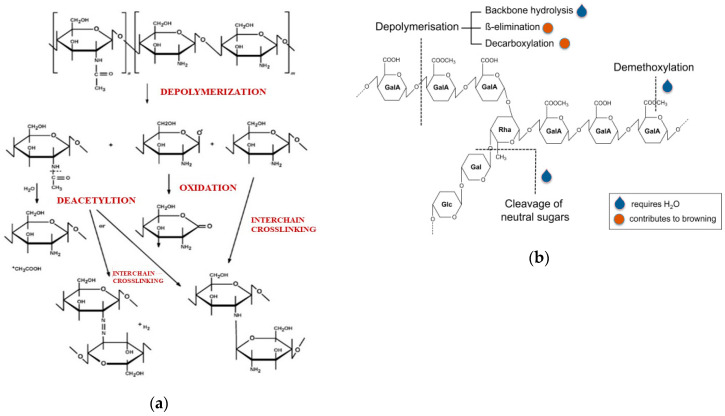
Depolymerization mechanisms of (**a**) chitosan [67] and (**b**) pectin [68].

Based on the mechanisms reported in Figure 5, the variations of these peak areas are related to the occurrence of a degradation process for the Ch:P blend. Hence, acquiring the total area of peaks in the range of 1700–1480 cm^−1^ and deconvoluting the curve in two peaks at around 1630 cm^−1^ (C=O stretching) and 1530 cm^−1^ (N–H bending), it is possible to profitably follow the degradation process of Ch:P blends, without and with additives.

**Figure 6:**
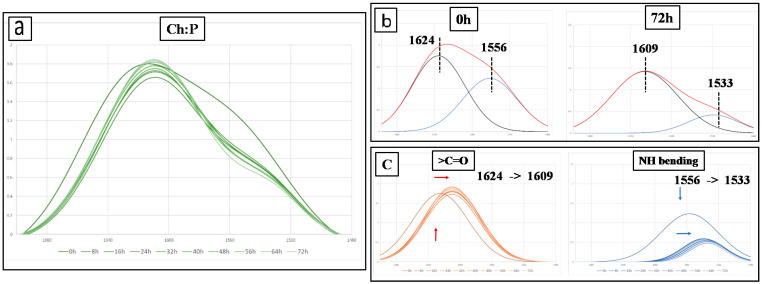
(**a**) Spectra in the range of 1700–1480 cm^−1^ at different exposure times of the Ch:P blend, (**b**) deconvolution of the peaks of Ch:P before exposure and at maximum exposure time, and (**c**) trends for the two deconvoluted peaks at different exposure time.

In Figure 6a, the complex peak in the range of 1700–1480 cm^−1^ for the Ch:P blend at different exposure times and (Figure 6b) the results of deconvolution of this complex peak before exposure (0 h) and at maximum exposure time (72 h) are plotted. Interestingly, in Figure 6c, the trends of deconvoluted two peaks as a function of exposure time are shown and it can be clearly noticed that (i) the peak at around 1630 cm^−1^ shifts toward lower wavelengths (from 1624 to 1609) and its intensity increases as a function of exposure time, and (ii) the peak at around 1530 cm^−1^ shifts again toward lower wavelengths (from 1556 to 1533), but its intensity decreases as a function of exposure time. Observed shifts for the two peaks can be related to a clear modification of the vibration mode for both >C=O and NH bending. It is worth noting that this phenomenon is well-pronounced at an early stage of exposure, i.e., after 8 h of exposure, and probably, it can be understood considering the loss of coordinated water molecules in the Ch:P structure and the occurrence of Ch:P structure changes.

**Figure 7:**
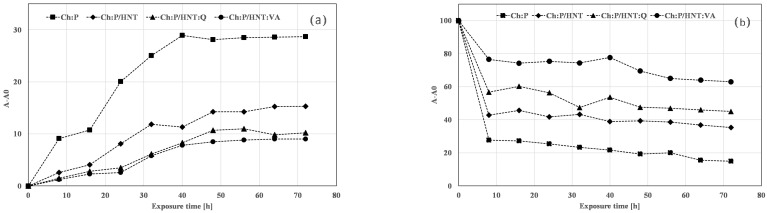
Variation of (**a**) >C=O and (**b**) NH bending for Ch:P and Ch:P/HNT without and with natural antioxidants.

Similar changes are observed also for the Ch:P samples containing HNT without and with antioxidant molecules, and in Figure 7a,b, the variations of >C=O and NH bending are plotted, respectively. It is worth noting that the presence of HNT leads to a decrease in the accumulation of >C=O groups and a reduced decrease of NH bending, and, moreover, this effect is exacerbated by the presence of both antioxidant molecules, see Figure 7a,b. According to the above reported degradation mechanisms, the observed trends in Figure 7a,b can be understand considering a fragmentation and reorganization of biopolymer chains of both biopolymers upon UVB exposure. This issue is less pronounced by the presence of HNT, and further slows down by HNT:Q and HNT:Q.

Therefore, the presence of HNT at this very high amount, i.e., 20% wt., plays an important role in the photo-oxidation resistance of films, particularly, HNT separately take the biopolymer macromolecules, and for this reason, the propagation of degradation phenomenon is disadvantaged. The presence of loaded Q and VA onto HNT further improves the photo-oxidation resistance of Ch:P, making them suitable as promising materials for cultural heritage protection.

## 4. Conclusions

In this research, bionanocomposite films based on natural Ch, P, HNT, and antioxidants, were successfully formulated through the solvent-casting technique. Therefore, the suitability of these materials as promising materials for cultural heritage protection was accurately investigated and assessed through different experimental analyses. 

The solubility of P into Ch was theoretically estimated by Hoy’s method, and based on performed calculations, the P is soluble into Ch. Based on the ζ-potential measurement, the null charge that corresponds to the matching between positive chitosan and negative pectin is obtained at a Ch:P mass ratio equal to 0.13.

The effect of the presence of HNT and HNT loaded with natural antioxidants (Q and VA) on the optical properties, wettability, mechanical behavior, and photo-oxidation resistance was investigated. Specifically, the optical properties and morphology of the bionanocomposite films are not negatively influenced by the presence of high amounts of HNT and HNT with natural antioxidants. It is worth noting that the presence of HNT generates a slight decrease of the water contact angle values, and additionally, it should be noted that the presence of Q and VA in the halloysite nanotubes does not further alter the film wettablility. The presence of HNT in the Ch:P blend leads to a slight decrease of the system rigidity, and interestingly, by adding HNT:Q and HNT:VA, the system rigidity is similar to that of an unfilled blend. Therefore, the HNT play a two-fold role—compatibilizer for the biopolymer blend and carrier for antioxidant molecules. The loaded antioxidant molecules exert protection action at the interface between the biopolymer matrix and inorganic HNT, making the bionanocomposite films increasingly resistant to the photo-oxidation. 

To sum up, all obtained results highlight that Ch:P/HNT, and even more Ch:P/HNT:Q and Ch:P/HNT:VA, can be considered promising materials for cultural heritage protection. 

## Figures and Tables

**Table 1:** Values of theoretical calculated solubility parameters of chitosan (Ch) and pectin (P).

Sample	*δ_tot_*, (J/cm^3^)^1/2^	*δ_p_*, (J/cm^3^)^1/2^	*δ_h_*, (J/cm^3^)^1/2^	*δ_d_*, (J/cm^3^)^1/2^
Chitosan (Ch)	29.54	17.99	18.71	14.10
Pectin (p)	28.26	17.26	17.57	13.85

**Table 2:** Assignment of main IR absorption bands of chitosan and pectin [24,25].

Chitosan		Pectin	
ν cm^−1^	Attribution	ν cm^−1^	Attribution
2927	symmetric C–H stretching	2929	symmetric-CH_2_ stretching
1736	>C=O stretching	1739	–O–C=O
1626	C=O of ion COO stretching of secondary amide group (amide I)	1634	–O^(−)^–C=O
1530	N–H bending (residue of amide II)	1015	–C–O–C–
1395	C=N stretching (amide III band)	955	rhamnogalacturonan (uronic acid)
1353	N–H in plan deformation	923	d-glucopyranosyl
955	piranose ring	890, 852	α-, β-glucosidic linkage
890	C–N fingerprint band	832	α-d-mannopyranose

**Table 3:** Measured contact angles of Ch:P and Ch:P containing HNT without and with natural antioxidants.

Sample	Left [°]	Right [°]	Average [°]
Ch:P	73.7	74.0	73.9
Ch:P/HNT	65.9	66.0	66.0
Ch:P/HNT:Q	68.7	67.3	68.0
Ch:P/HNT:VA	68.6	68.3	68.5

## Supplementary Materials

The following are available online at https://www.mdpi.com/2073-4360/12/9/1973/s1, Figure S1; Thermogravimetric curves (TGA) for HNT (black line), HNT/VA (red line) and HNT/Q (yellow line), Figure S2: Optical microscopy images for: (a) Ch:P blend at mass ratio 0.13; (b) Ch:P blend at mass ratio 1, Figure S3: FTIR spectra of Ch:P at different exposure time, Figure S4: FTIR spectra of Ch:P/HNT at different exposure time, Figure S5: FTIR spectra of Ch:P/HNT:Q at different exposure time, Figure S6: FTIR spectra of Ch:P/HNT:VA at different exposure time
