# Supplementary material for: Bionanocomposite Films Containing Halloysite Nanotubes and Natural Antioxidants with Enhanced Performance and Durability as Promising Materials for Cultural Heritage Protection
Source: Polymers (Basel). 2020 Aug 31;12(9):1973. doi: 10.3390/polym12091973 (PMC7564337; doi:10.3390/polym12091973)
Supplement: Supplementary file 1 [file polymers-12-01973-s001.pdf]

Supplementary Figures

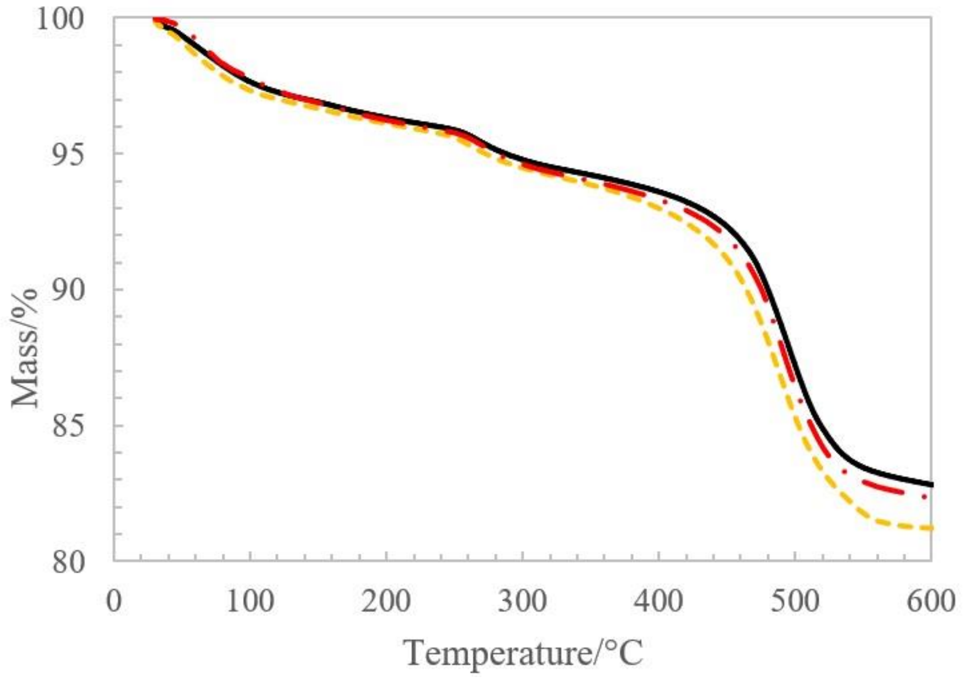

Figure S1. Thermogravimetric curves (TGA) for HNT (black line), HNT/VA (red line) and HNT/Q (yellow line)

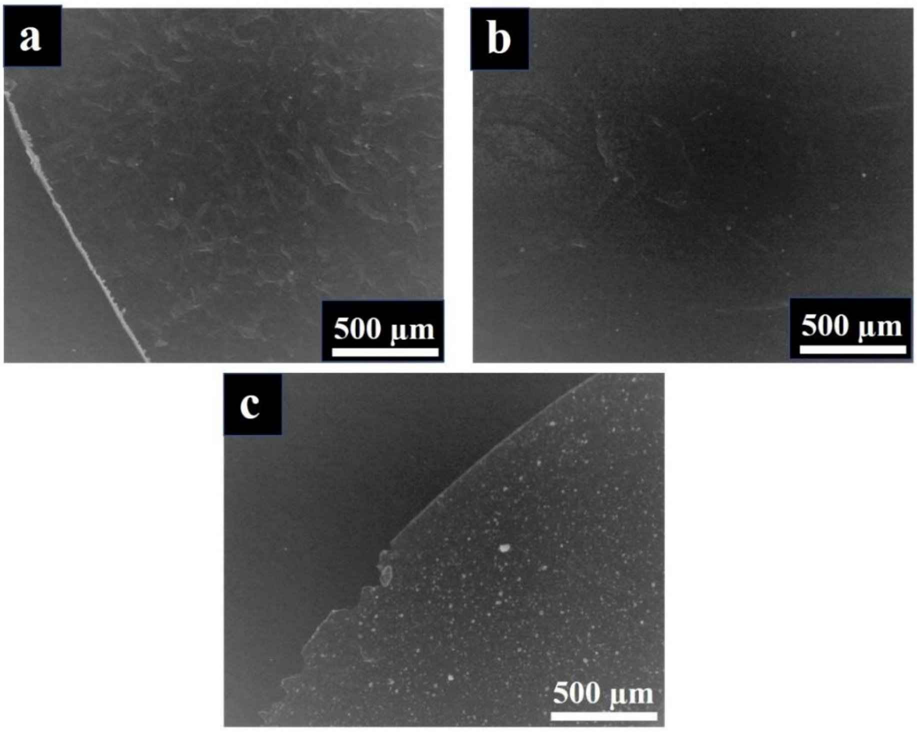

Figure S2. Optical microscopy images for: (a) Ch:P blend at mass ratio 0.13; (b) Ch:P blend at mass ratio 1; (c) Ch:P blend at mass ratio 1 with 20 wt% of HNTs.

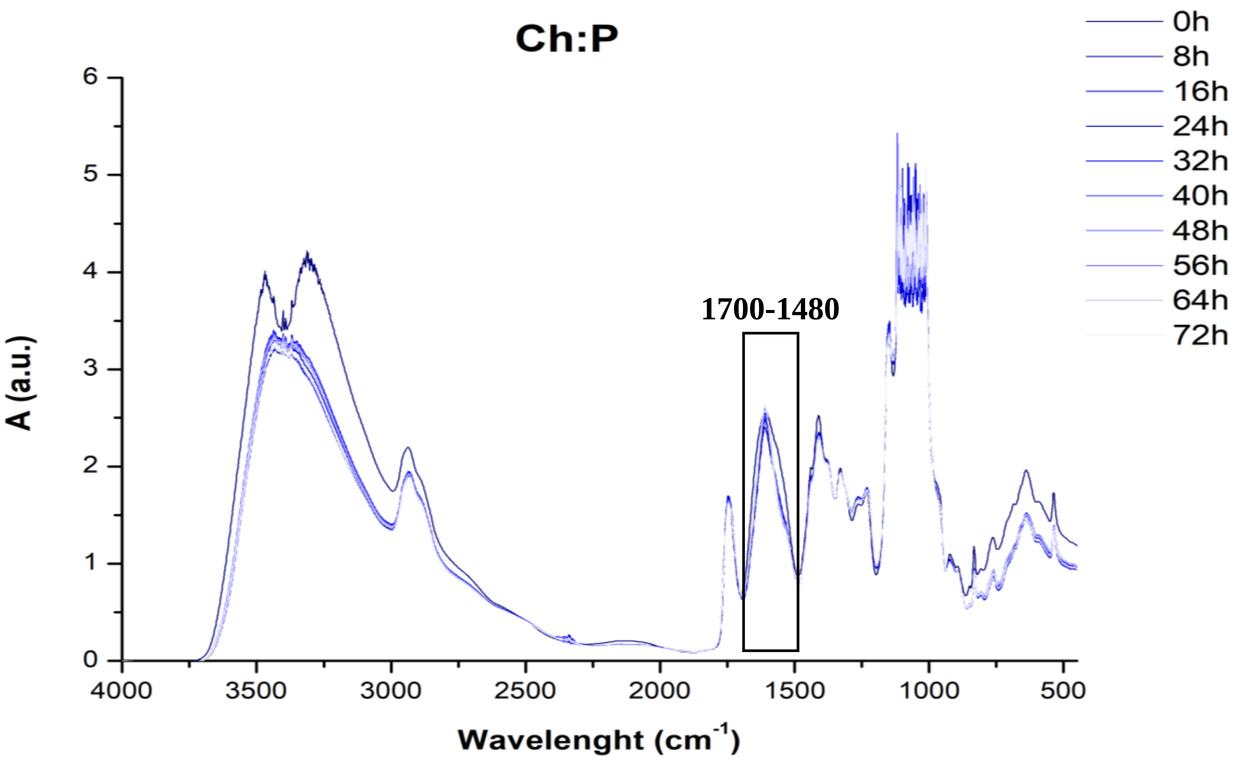

Figure S3. FTIR spectra of Ch:P at different exposure time

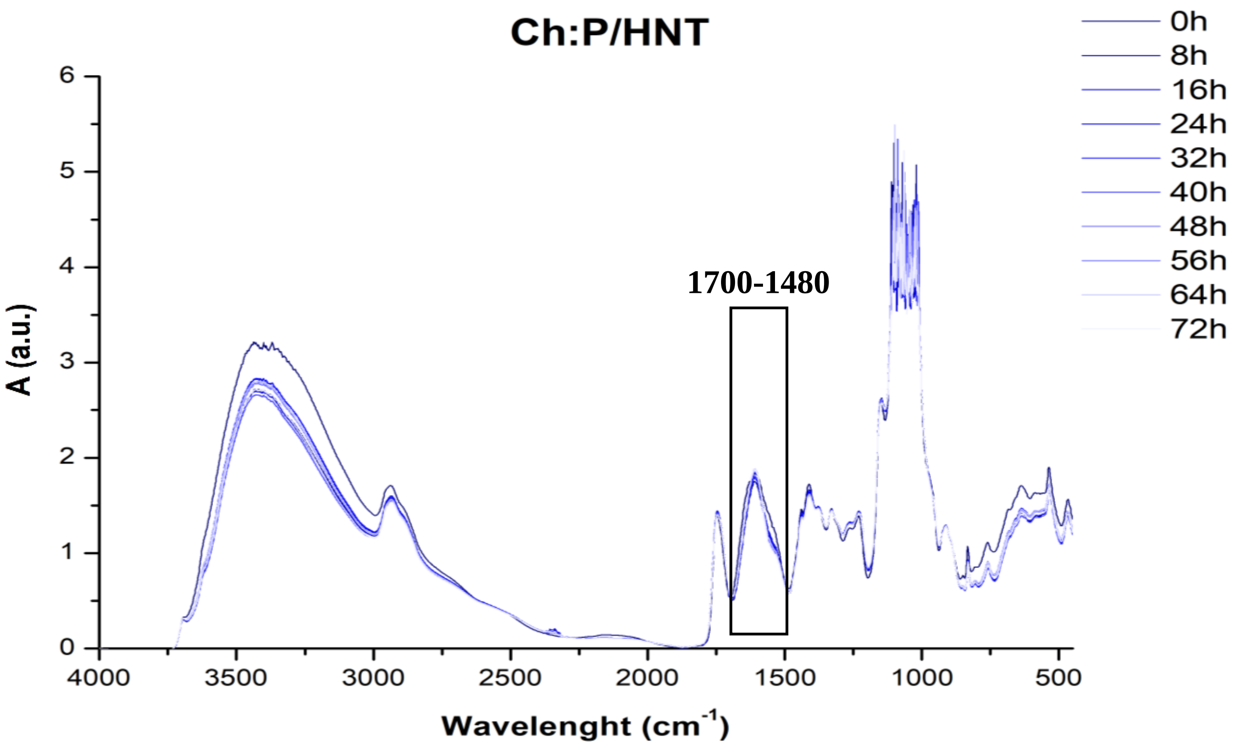

Figure S4. FTIR spectra of Ch:P/HNT at different exposure time

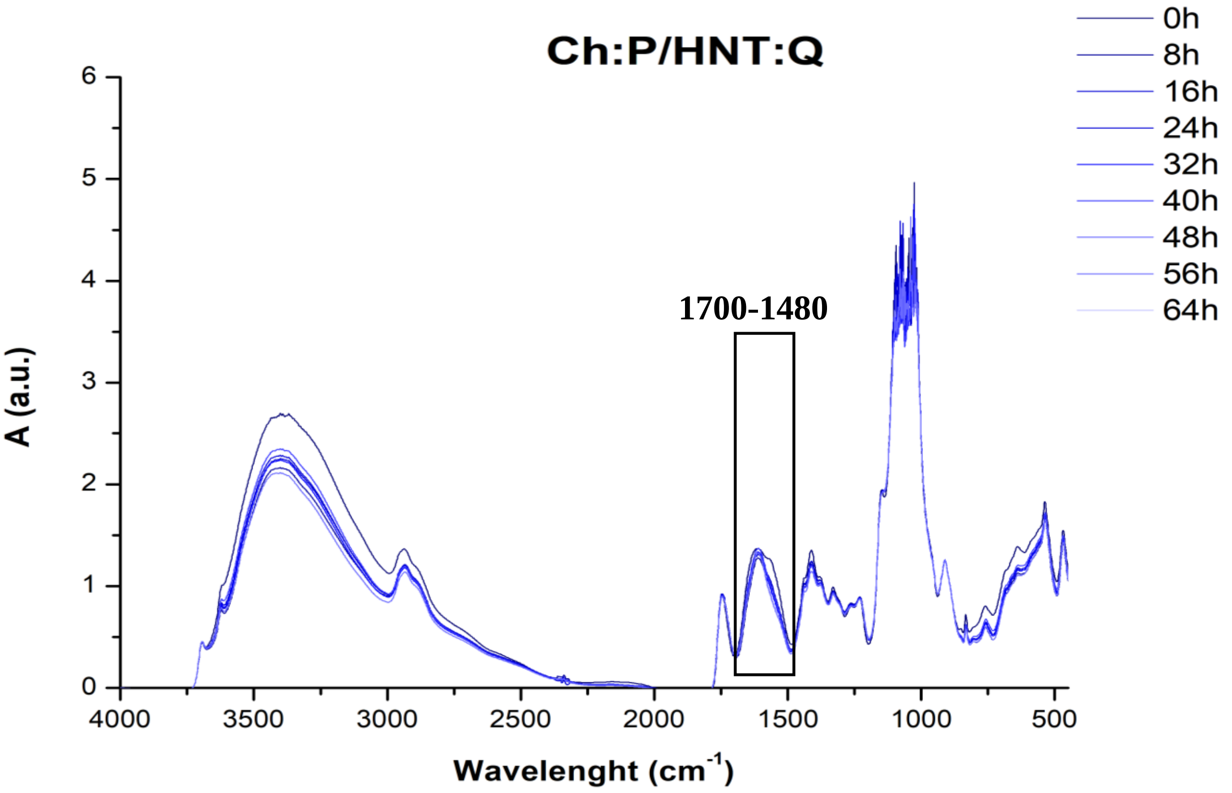

Figure S5. FTIR spectra of Ch:P/HNT:Q at different exposure time

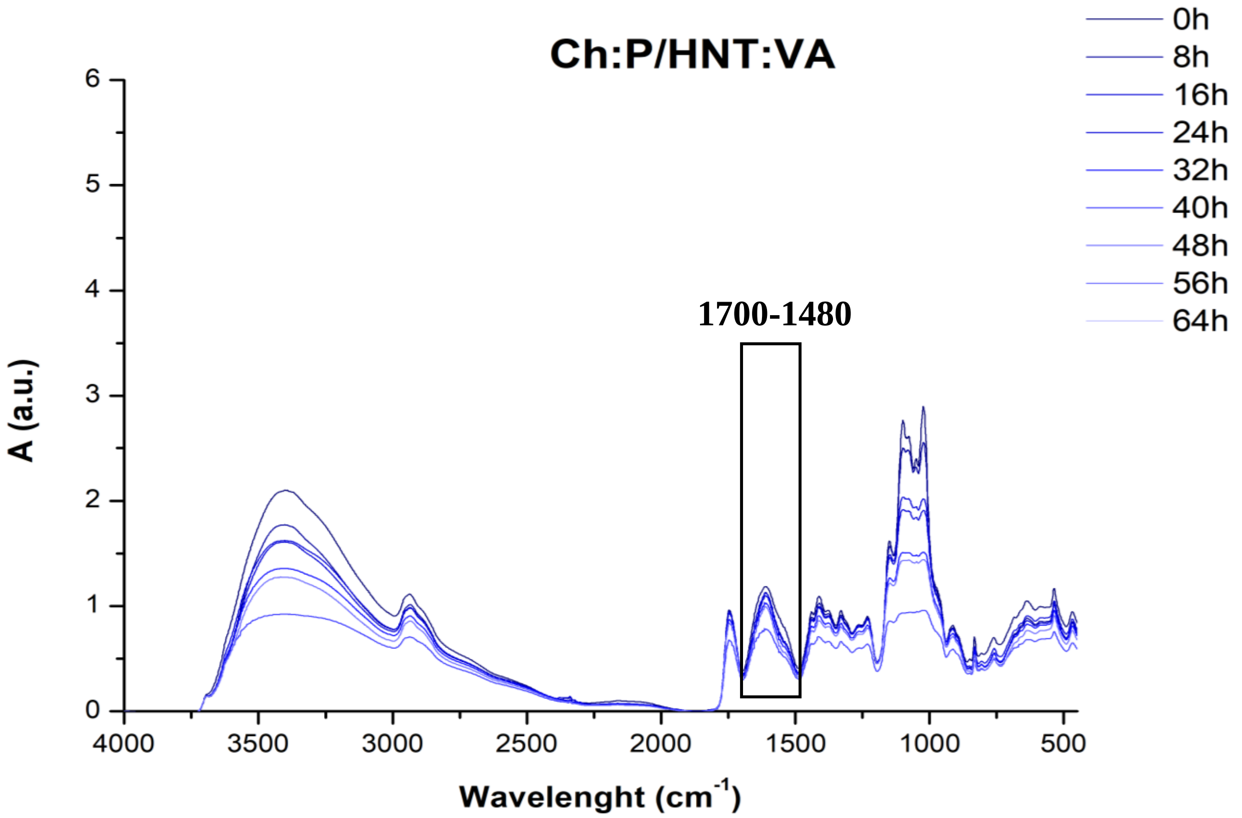

Figure S6. FTIR spectra of Ch:P/HNT:VA at different exposure time
